# Diagnostic waste: whose responsibility?

**DOI:** 10.1186/s12992-022-00823-7

**Published:** 2022-03-12

**Authors:** Alice Street, Eva Vernooij, Mohamed Hashim Rogers

**Affiliations:** 1 School of Social and Political Science, University of Edinburgh, Edinburgh, UK; 2 Department of Microbiology, College of Medicine and Allied Health Sciences, University of Sierra Leone, Freetown, Sierra Leone

**Keywords:** Diagnostics, Waste management, Sustainability, Responsibility, Product development

## Abstract

Waste management is notably absent from current discussions about efforts to improve access to diagnostics in low-and middle-income Countries (LMICs). Yet an increase in testing will inevitably lead to an increase in diagnostic waste, especially since many of the diagnostic tests designed for use in LMICs are single-use point-of-care tests. Diagnostic waste poses a threat to both human and environmental health. In this commentary we draw on our experience of diagnostic waste management in Sierra Leone and review current evidence on: the volume and impact of diagnostic waste in LMICs, existing health-care waste management capacity in LMICs, established national and international policies for improving health-care waste management, and opportunities for strengthening policy in this area. We argue that questions of safe disposal for diagnostics should not be an afterthought, only posed once questions of access have already been addressed. Moreover, responsibility for safe disposal of diagnostic waste should not fall solely on national health systems by default. Instead, consideration of the end-life of diagnostic products must be fully integrated into the diagnostic access agenda and greater pressure should be placed on manufacturers to take responsibility for the full life-cycle of their products.

## Background

‘Discard sample and assay waste according to your local safety regulations.’ So states the standard small print found at the bottom of countless manufacturer labels for single-use diagnostic products. But what if no local or national guidelines or regulations exist? Or what if those guidelines exist on paper, but there is no infrastructure, systems, or workforce in place to enable health facilities to adhere to them? Since the start of the COVID-19 pandemic, the diagnostics pillar of the Access to COVID-19 Tools (ACT) Accelerator partnership has procured over 116.9 million tests for use in low-and-middle-income countries (LMIC), of which 43.2 million were polymerase chain reaction (PCR) tests and 73.7 million antigen-detecting rapid diagnostic tests (Ag-RDT) [20]. This achievement represents valuable progress in efforts to improve global access to life-saving diagnostic tools in the pandemic. But with the global health community’s attention focused on questions of diagnostic access, far less consideration has been given to the end-life of diagnostic products [13, 15].

Cartridge-based polymerase chain reaction (PCR) tests and Ag-RDT tests [lateral flows] used to detect the SARS-CoV-2 virus are single-use devices. Their self-contained, easy-to-use format makes them especially promising for the extension of testing to places with limited laboratory infrastructure, and for the decentralisation of community testing. Yet, each one of those 116.9 million tests has also generated plastic, infectious, and potentially toxic waste that must be disposed of safely after use. Diagnostic waste poses multiple threats to human and environmental health [21, 24]. Ag-RDT cassettes are made from petroleum-derived plastics, which have a large CO_2_ footprint and are non-biodegradable. A recent study estimated that PCR testing for COVID-19 generated 15,000 tons of plastic waste globally to August 2020 [5]. A World Health Organization (WHO) report on COVID-19 related healthcare waste calculated that test kits procured solely through the United Nations (UN) supply portal since the start of the pandemic had the potential to generate 2,600 tonnes of general waste (mainly plastic) [32]. But the problem of diagnostic waste goes well beyond the issue of single-use plastics.

Needles used to draw blood specimens pose a significant infection threat to the people who handle them [16]. Recent research has found that up to 75% of healthcare waste workers have sustained some kind of sharps-related injury [23]. Spent Ag-RDTs and PCR cartridges contain potentially infectious materials and harmful reagents. PCR test cartridges contain guanidine thiocyanate (GTC), which is highly toxic, and can generate dangerous gases when mixed with cleaning agents [14]. The WHO calculates that test kits procured through the UN portal since the beginning of the pandemic has the potential to generate 731,000 L of chemical waste [32]. When incinerated at low temperatures, diagnostic waste of all kinds can generate emissions of dioxins, furans, and particulate matter that are harmful to human health [24]. The disposal of chemical liquid waste in landfills and public sewage systems can lead to the contamination of groundwater and drinking water (ibid).

In many LMICs, neither the regulatory frameworks nor the physical infrastructure and expertise required are in place to enable the safe disposal of diagnostic waste [6, 10, 14,22,33]. A recent report by the WHO and the United Nations Children’s Fund (UNICEF) found that, in 2016, more than half the countries in Sustainable Development Goal regions that had data available lacked basic healthcare waste management services [33]. The WHO’s most recent Global Progress Report on Wash in Health Care Facilities reported that one of three health care facilities globally do not segregate waste safely [29].

So where has all the waste that has been generated by COVID-19 testing gone? In this commentary, we contextualise findings from research on diagnostic waste flows in Sierra Leone in current evidence around diagnostic waste management in LMICs, COVID-19 diagnostic procurement, and wider debates around diagnostic access in global health. We argue that questions of sustainability and responsible waste management are a notable omission from efforts to improve access to diagnostic services in LMICs, and that this results in responsibility for waste management falling almost entirely on the recipients of diagnostic products. We argue that improving sustainability is central to diagnostic access and should not be treated as an afterthought. This requires a rethink of how responsibility for waste flows is distributed across the product life-cycle, and draws attention to the role of corporate actors in the production of hazardous and unsustainable materials.

## Failing to learn from previous epidemics

Our experience of diagnostic waste in Sierra Leone in the aftermath of the 2014–16 Ebola outbreak in West Africa provides some possible pointers as to where waste from COVID-19 testing is ending up. In 2019, three years after the last confirmed Ebola case in the country, we carried out ethnographic and survey research in the capital Freetown and in the neighbouring Western Area administrative subdivision in order to assess the availability of infrastructure and systems for disposing of diagnostic waste. We found that many of the measures taken during the Ebola outbreak and in its immediate aftermath to strengthen biosafety and waste management systems had already begun to falter.

In community health centres (CHC), staff recalled attending biosafety trainings run by international agencies during the Ebola outbreak, but few had since received refresher courses. Many of the incinerators installed during the outbreak and funded by donors had fallen into disuse, either because of faults in their construction or a lack of funds for fuel. Some incinerators had never even been used because the administrative process for transferring ownership from the donor to the government had never been completed. Of a total of 40 CHCs in Western Area, fewer than half had a functioning incinerator at the time of our visit.

**Fig. 1:**
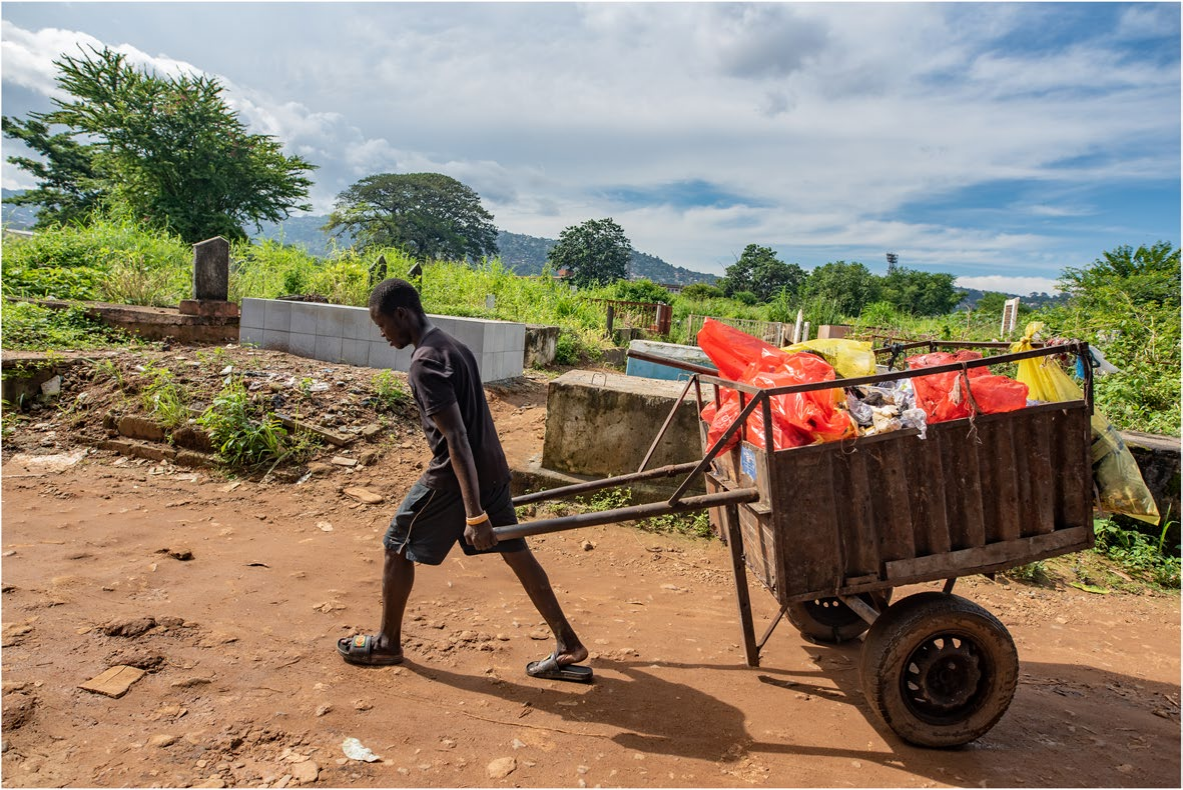
Waste collector carts medical waste from Community Health Post to public dump, Western Rural Area, Sierra Leone. Credit: Olivia Acland/DiaDev

**Fig. 2:**
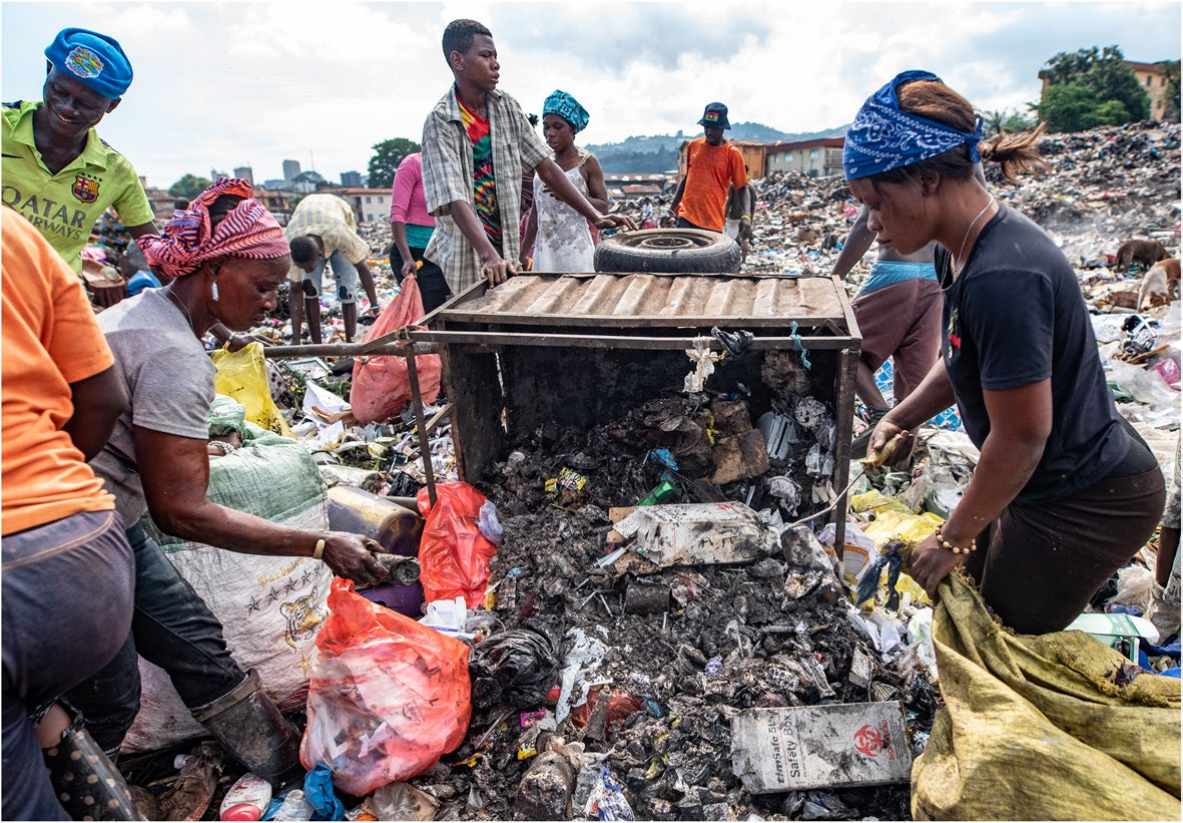
People at public rubbish dump pick through the medical waste from a Community Health Post (CHC), Western Rural Area, Sierra Leone. Credit: Olivia Acland/DiaDev

So where else did diagnostic waste go? Sometimes infectious waste was openly burned in the grounds outside the CHC. A third of facilities had burial pits, but in many cases the construction was makeshift and did not comply with guidelines on depth. In many facilities health workers arranged informal waste collections by *keke* [tricycle] drivers or people using wheelbarrows. Waste that had previously been segregated into ‘general’ and ‘infectious’ categories in the laboratory was then recombined by the waste collectors outside the facilities before being transported to public dumping sites (Fig. 1). At the public dumps we observed people, among them children, sorting through medical waste, including the contents of sharps boxes, by hand (Fig. 2).

**Fig. 3:**
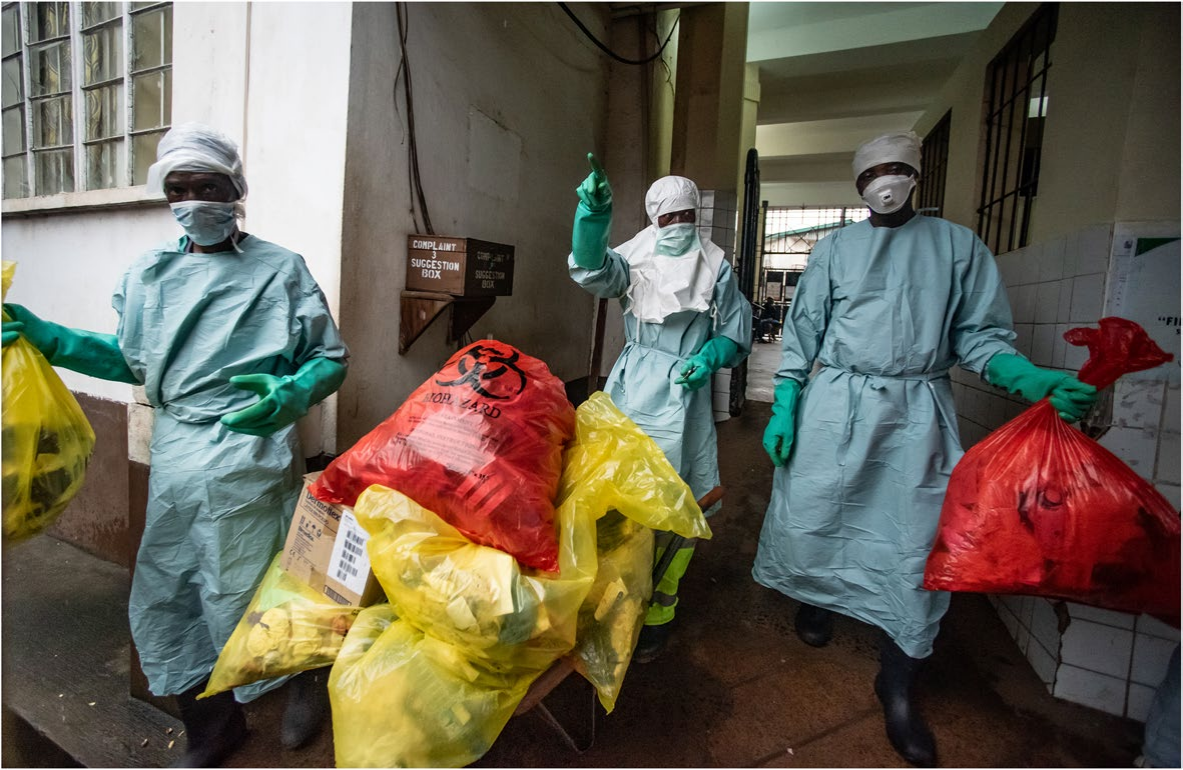
Cleaner takes wheelbarrow full of hospital waste to the incinerator, Freetown, Sierra Leone. Credit: Olivia Acland/DiaDev

**Fig. 4:**
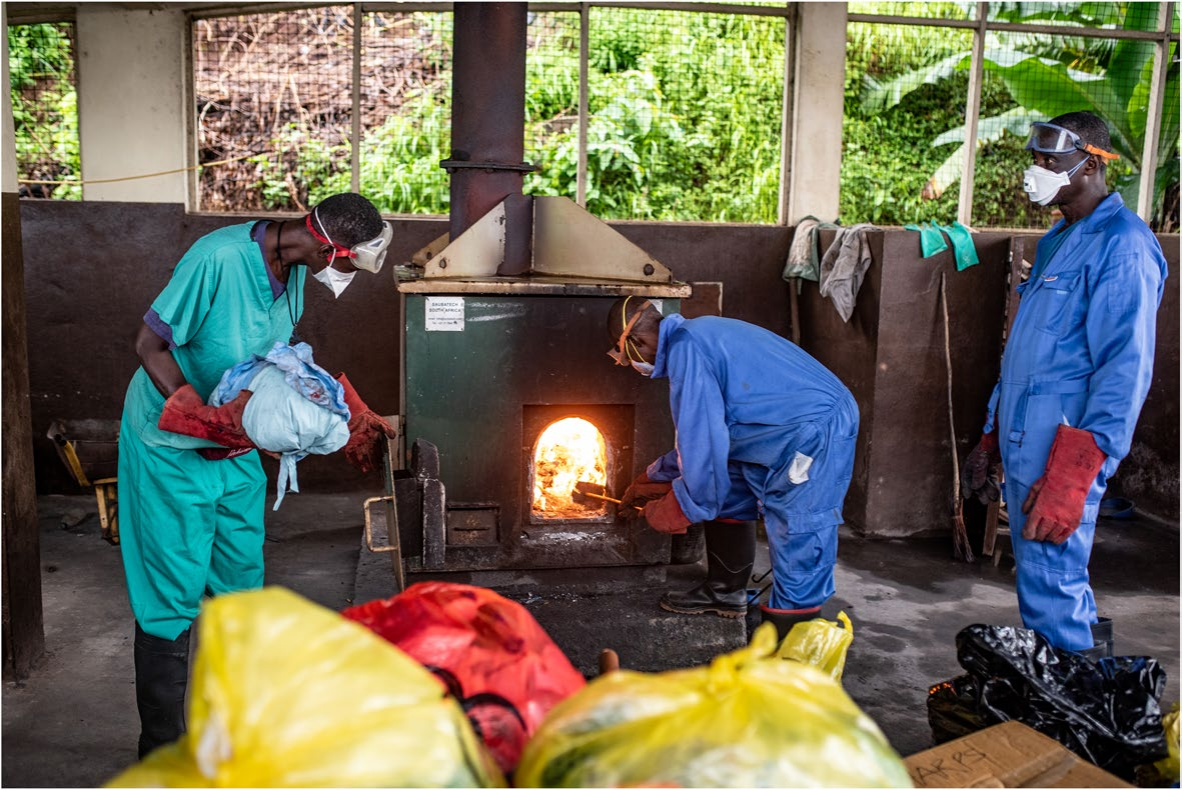
Incinerator room in government hospital, Freetown Sierra Leone. Credit: Olivia Acland/DiaDev

The situation at a government referral hospital in Freetown was little better. Here, externally contracted cleaning staff transported infectious waste from the laboratory, wards, or interim transit areas to the incinerator in uncovered wheelbarrows (Fig. 3). Frequent delays in collection from transit areas resulted in spillages and encouraged scavenging by dogs and vermin. Highly infectious waste, including used sharps, liquid waste from diagnostic machines, blood collection tubes, spent cartridges, reagents, and Ag-RDTs, are supposed to be transported in clearly labelled red bags, but a frequent shortage of these forced staff to use bags labelled for general waste instead. The hospital incinerator was too small to accommodate all the infectious waste generated by the hospital, and the staff burned large volumes of waste in the open area behind the patient wards, which sent up dark plumes of smoke (Fig. 4). Liquid waste was poured down a pipeline that led directly to the public beach, where we also found remnants of other laboratory waste, including needles and test tubes. Unsurprisingly, morale among cleaners and incinerator staff was low. Pay was little, there were few career opportunities, and staff received scant recognition for doing their job well. Cleaners and incinerator staff lacked sufficient or effective protective clothing, and we were told that one staff member had been hit in the eye by an incinerator blast and had later died from the injury. These findings corroborate the findings of an audit of healthcare waste in Sierra Leone undertaken in 2018 [2].

The ACT Accelerator diagnostics pillar aims to increase testing in African countries to 40 million tests a month in 2022 [18]. Ensuring that African countries get access to global diagnostic supplies is vital to address current inequities in the COVID-19 response and enable the continent to get ahead of the pandemic [5]. But if the waste management systems that are in place to respond to this increased volume of tests are anything like those that we observed in Sierra Leone in 2019, as is suggested to be the case by WHO reporting and a recent review of waste management systems in 11 African countries [14, 29, 32], then we are heading for a diagnostic waste crisis. This is even more so the case when the additional waste generated by the distribution of personal protective equipment (PPE) and the vaccine rollout is taken into account  [3, 7, 9, 22].

## More tests means more waste

The challenges associated with the disposal of diagnostic waste in LMICs will evidently increase with improved availability of point-of-care diagnostic tests, and yet questions of waste management are notably absent from the current conversation about access. The recent Lancet Commission on diagnostics makes ten recommendations to address the diagnostic gap in LMICs [9]. These include making point-of-care tests for key conditions available at all primary health centres—in line with the WHO’s Essential Diagnostics List— [31] and strengthening governance and regulatory frameworks to support and oversee diagnostic quality and safety. But it makes no mention of the challenges that decentralised testing with single-use devices poses for waste disposal, or the urgent need for investment in waste management infrastructures in parallel with improving access to diagnostic products.

The global health community has been aware of the risks associated with single-use point-of-care devices for some time. The WHO’s 2010 roadmap for rollout of the GeneXpert test for tuberculosis testing acknowledged that ‘waste generated by tests run on the platform was considerably more than for microscopy’ [26]. A 2010 diagnostic innovations map noted that trade-offs for point-of-care tests include ‘more waste from packaging and disposables’ [4]. More recently, research on HIV viral load testing showed that the testing process was generating close to a million litres of effluent waste annually [12]. But far too often, advocacy for access to diagnostics has sidelined questions of waste management, viewing the issue as something to be dealt with only after the desperately needed tools have arrived.

## Who is responsible for diagnostic waste?

Currently, responsibility for diagnostic waste falls on national governments by default. The WHO’s 2017 global model regulatory framework for medical devices states that: ‘For disposable devices, decontamination and proper waste management practices according to the manufacturer’s instructions should be required’ [28]; [22] meanwhile, manufacturer guidelines, as noted above, usually simply direct users to follow ‘local guidelines’.

Since publishing in 1999 the first handbook for the safe management of waste from healthcare activities, the WHO has provided valuable guidance and support to national governments to strengthen regulation and policies for waste management [25]. The LabCoP forum, which is supported by the African Society for Laboratory Medicine (ASLM) also provides an important platform for the sharing of tools and resources to guide monitoring and strengthening of waste management systems in LMICs [1, 14]. Recent efforts by The Global Fund to address issues of environmental sustainability and responsibility have likewise focused on local infrastructure and systems by encouraging applicants to incorporate measures to improve healthcare waste management into their funding proposals [21].

But what about the industry that develops and manufactures point-of-care diagnostic products? Odhiambo and his co-authors suggest that manufacturers might contribute to the costs of waste management through corporate social responsibility programmes [9]. But such an approach both accepts that the role of manufacturers in waste management is voluntary and charitable (rather than obligatory) and takes for granted the current levels of harmful waste generated by diagnostic products. With the focus of the global health community on how to incentivise industry to develop diagnostic tests for use in LMICs, very little is being done to put pressure on manufacturers to improve the safety and sustainability of their products. There is legitimate concern that the search for greener and safer products might increase manufacturing costs and thus make diagnostics less affordable. But the failure to build the costs of waste management into the market price for products only pushes those costs onto the institutions and people who manage their disposal at the point of use.

Is it right that responsibility for the disposal of products should fall solely on the shoulders of the countries that consume them? In other sectors, responsibility for disposal of single-use products is increasingly being shared across the supply chain. In the e-waste sector, for instance, the concept of extended producer responsibility (EPR) is gaining traction. EPR places responsibility on the manufacturer for the environmental and health impact of a product across its entire life cycle. The Global Fund’s recent technical brief on sustainable healthcare management mentions ‘producer responsibility’ as a guiding principle for implementing a systems approach to healthcare waste management, and emphasises the role of responsible procurement in preventing the generation of harmful and polluting waste [25]. But the brief places responsibility for the implementation of these principles on applicants, which in many cases will mean national governments.

How then might greater responsibility for the generation and disposal of waste also be placed on manufacturers? One starting point is target product profiles (TPP). These guidance documents are published by international health agencies and used to brief developers and manufacturers about the technical specifications that funders and policymakers expect from future diagnostic devices in a specified area. The TPP released by the WHO in October 2014 for the *Zaire ebolavirus*, intended to be ‘a simple test to be used in the control of the Ebola outbreak in West Africa’, and aimed at incentivising industry investment in Ebola diagnostics, did not provide any dedicated guidance on acceptable features for waste disposal [27]. And yet, when we spoke in 2019 to WHO representatives who had been involved in the Ebola response, they cited the lack of waste management infrastructure as one of the key reasons why rapid antigen tests that received emergency listing through the WHO’s Emergency Use Assessment Listing mechanism (EUAL) were not widely deployed in the region [11].

Some progress has since been made. The TPP for a highly accurate confirmatory test for SARS-COV-2, which was published by the WHO in 2020, includes specific recommendations for waste management [30]. The TPP lists ‘standard biohazardous waste disposal or incineration of consumables, no high temperature incineration required’ as an ‘acceptable’ approach to associated waste management. It lists ‘[a] small environmental footprint; recyclable or compostable plastics for test cartridges and other materials after decontamination, no incineration required’ as ‘desirable’. Nonetheless, a different TPP for a SARS-CoV-2 point-of-care tests for use where a reference assay is not available lists the generation of ‘routine biohazard waste’ as ‘acceptable’ and includes no ‘desirable’ characteristics for these devices at all. This lack of consistency highlights the insufficient consideration that is being afforded to waste management from the very first stages of diagnostic design.

## Conclusion

The lack of ambition for improved waste management espoused by current TPPs for diagnostic tests does not reflect what is technically possible. Significant advances are being made in green chemistry, [28] and a few standout companies are already developing biodegradable, reusable, or recyclable plastic housings for rapid diagnostic tests [8]. With more encouragement and incentive, along with higher expectations being placed on them, many more companies could be doing the same. Multiplexing—the development of tests that serve more than one disease—is increasingly recognised as important for effective disease management in resource-limited settings, but also has the potential to significantly reduce volumes of diagnostic waste [17]. Diagnostic waste is not only generated at the site of use. It is also built into products at the point of design. This present failure to consider waste management at the design stage for point-of-care tests is all the more striking given that the whole point of these devices is that they are meant to be deployable anywhere. Why is it that some infrastructural limitations, such as the lack of laboratory equipment or expertise, are incorporated into the design brief by diagnostic developers while others, such as the lack of waste management regulations or functioning incinerators, are not?

In a moment when African countries are struggling to access enough diagnostic tests, it is difficult to prioritise resources for their safe disposal. But without an accompanying focus on the end of the product lifecycle, the push to increase the availability of diagnostic products for COVID-19 and other priority diseases in LMICs risks significant harm to human health and the environment. In this respect the current push to increase regional manufacturing in Africa of diagnostic tests is also an opportunity to invest in the manufacture of tests that are truly appropriate for use in the places where they will be deployed—including by taking into consideration the existing waste management infrastructures. Yes, we need more investment in national waste management infrastructures, regulatory frameworks, and workforce training. But the companies that stand to benefit from the growth of the diagnostics market in the coming years also share responsibility for the afterlives of those products.

## Data Availability

The survey data reported in this paper is openly available at https://doi.org/10.7488/ds/3401.

## Abbreviations

Ag-RDT: Antigen-detecting rapid diagnostic tests
CHC: Community Health Centre
EPR: Extended producer responsibility
EUAL: Emergency Use Assessment Listing
LMIC: Low-and Middle-Income Country
PCR: Polymerase chain reaction
PPE: Personal protective equipment
TPP: Target product profile
UN: United Nations
UNICEF: United Nations Children’s Fund
WHO: World Health Organization
